# Innovative approaches to neonatal jaundice diagnosis and management in low-resourced settings

**DOI:** 10.4102/safp.v66i1.5833

**Published:** 2024-02-29

**Authors:** Haroon Saloojee

**Affiliations:** 1 Department of Paediatrics and Child Health, Faculty of Health Sciences, University of the Witwatersrand, Johannesburg, South Africa

**Keywords:** neonatal jaundice, hyperbilirubinaemia, bilirubin, phototherapy, resource-constrained

## Abstract

Persistent challenges in addressing severe neonatal hyperbilirubinaemia in resource-constrained settings have led to ongoing and often unacceptable rates of morbidity, disability and mortality. These challenges stem from limitations such as inadequate, inefficient or financially inaccessible diagnostic and therapeutic options. However, over the past decade, noteworthy innovations have emerged to address some of these hurdles, and these innovations are increasingly poised for broader implementation. This review provides a concise summary of these novel, economically viable diagnostic solutions, encompassing point-of-care assays and smartphone applications, as well as treatment modalities, notably more effective phototherapy and filtered sunlight. These advancements hold promise and have the potential to meaningfully reduce the burden of neonatal hyperbilirubinaemia, signifying a promising shift in the landscape of neonatal healthcare.

## Introduction

Jaundice is a common neonatal condition that occurs in about 60% of term and 80% of preterm infants in the first week of life.^1^ It is characterised by a physiological elevation of serum bilirubin, peaking between days 3 and 5 of life and returning to normal values within approximately 2 weeks. While most increases in bilirubin are benign, about 10% of term and 25% of preterm infants may develop severe hyperbilirubinaemia,^2^ leading to acute bilirubin encephalopathy and kernicterus.

These complications are preventable if worsening hyperbilirubinaemia is identified early and treated promptly. Severe hyperbilirubinaemia has different aetiologies, some related to genetic traits and geographical location. Serious complications of neonatal hyperbilirubinaemia, including neurological disability and death, occur more frequently in low- and middle-income countries (LMICs).^3^ This is both the result of risk factors for severe hyperbilirubinaemia occurring more frequently in these settings, such as disorders leading to haemolysis and higher sepsis rates, as well as management challenges, including low recognition of jaundice by caregivers and healthcare professionals and inadequate or unaffordable diagnostic and therapeutic options.

In the past decade, innovations addressing some of these challenges have emerged that may be affordable and potentially scalable in resource-constrained settings (RCS). This article focuses on the two most prevalent challenges in neonatal jaundice diagnosis and management – an inability to measure total serum bilirubin (TSB) and inadequate or inaccessible phototherapy. It highlights innovative solutions and strategies developed to address each.

## Diagnosis

Most healthcare providers in RCS rely on visual inspection of the skin and sclera for jaundice screening. Visual inspection, while helpful for excluding jaundice, is imprecise for assessing the severity of jaundice and determining the need for therapy. Serum bilirubin is the gold standard test for diagnosing neonatal jaundice and is essential for the definitive diagnosis of severe hyperbilirubinaemia and treatment decisions. It requires blood sampling from the newborn and laboratory processing making it inaccessible in most doctors’ offices and primary healthcare centres. Health facilities in RCS often experience lengthy delays in obtaining TSB results. Furthermore, serum bilirubin usually peaks on day 4 of life, long after most newborns are discharged home. New strategies to address this deficiency include the use of point-of-care bilirubin assay testing, transcutaneous bilirubinometry (TcB), icterometers and smartphone digital camera-based bilirubin estimation (Table 1).

**TABLE 1:** Summary of diagnostic modalities available for neonatal hyperbilirubinaemia detection.

Name	Sample processing time (min)	Bilirubin measurement range (µmol/L [mg/dL])	Diagnostic accuracy (Outcome: indicators)	Device cost (USD[$]/Rand)†	Cost per test (USD[$]/Rand)†
**Bilirubinometer**					
BiliDx^®^	5–10	17–684 (1–40)	Error grid analysis: 97% of BiliDx^®^ measurements resulted in the same clinical decision as TSB result	$1122.00 (R20 196.00)	Unavailable
Bilistick^®^	2	17–684 (1–40)	Jaundice requiring phototherapy: Sens: 71%; Spec: 99%; +LR 48.1	R76 676.00	R150.00
BiliSpec	1	-	BiliSpec correlated well with TSB (*r* = 0.996).	$100.00 (R1800.00) (estimate)	$0.10 (R1.80)
Transcutaneous	Immediate	17–340 (1–20)	‘Significant hyperbilirubinaemia’ Sens: 74% – 100%; Spec: 18% – 89%	R50 000.00 – R100 000.00	Nil
**Icterometer**					
BiliStrip™	Immediate	85–235 (5–14)	Jaundice requiring phototherapy: Sens: 96%; Spec: 23%; +LR: 1.2	Unavailable	Nil
Bili-ruler™	Immediate	188 to > 340 (11 to > 20)	TSB ≥ 222 µmol/L Sens: 82%; Spec: 74%; +LR 3.2	$1.00 – $5.00 (R18.00 – R90.00)	Nil
**Smartphone digital camera-based bilirubinometer**					
BiliCam	Immediate	-	TSB ≥ 291 µmol/L: Sens: 100%; Spec: 76%; +LR: 4.2	Unavailable	Nil
Picterus	Immediate	-	TSB > 250 µmol/L: Sens: 100%; Spec: 69%; +LR: 3.1	Unavailable	Nil
Biliscan	Immediate	-	Jaundice requiring phototherapy: Sens: 77%; Spec: 71%; +LR: 2.7	Unavailable	Nil

+LR, positive likelihood ratio; Sens, sensitivity; Spec, specificity; TSB, total serum bilirubin.

† , Assumption: $1.00 = R18.00

### Point of care testing bilirubinometry

#### BiliDx^®^

BiliDx^®^ is a low-cost, lateral flow cassette device for point-of-care bilirubin measurement. Following a heel stick in the neonate, capillary blood is collected via a plastic transfer pipette, and the sample applied to a port on the BiliDx^®^ cassette. The cassette is inserted into the BiliDx^®^ device, with a TSB reading appearing on the screen in a few seconds. No reagent is required. In a study conducted in central hospitals in Malawi and Nigeria, the device performed well compared to a reference laboratory standard.^4^ Error grid analysis showed that 97% of samples measured with BiliDx^®^ would have resulted in the same clinical decision as when the reference bilirubin standard was used, exceeding the 90% accuracy of concomitant TcB.

**FIGURE 1:**
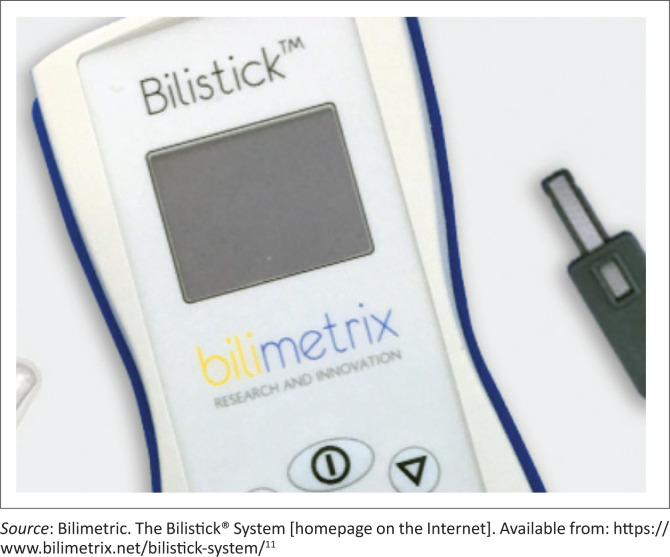
Bilimetrix Bilistick point of care assay device.

The *Bilistick* system is another point-of-care bilirubin assay. It consists of a Bilistick^®^ Test Strip, requiring one microdrop of blood and a Bilistick^®^ Reader that analyses the sample. Results are obtained in 2 min. Simplification of the calibration process and improved clinical accuracy of the device are required (Figure 1).^5^ *Bilispec* is another point-of-care device currently undergoing study. It promises to be affordable at a manufacturing cost of under $100.00 and a per-test cost of the lateral flow strip of less than $0.10^6^.

#### Transcutaneous bilirubinometry

Transcutaneous bilirubinometry devices have become routine screening tools for neonatal jaundice in well-resourced settings. Transcutaneous bilirubinometry relies on optical spectroscopy, where the degree of light absorption by bilirubin is connected to its concentration in the skin. The advantage of this screening method is that it is non-invasive, painless, quick and user friendly. In RCS, TcB is infrequently available in public healthcare settings and still relatively expensive (about R50 000.00 – R100 000.00 in South Africa).^7^ There is conflicting evidence regarding the sensitivity of these devices in pigmented babies as darker skin hues may potentially confound light reflectance.^8^ The sensitivity of various TcB cutoff values to detect significant hyperbilirubinaemia ranges from 74% to 100% and specificity from 18% to 89%.^9^ These instruments may overestimate TSB at low values and underestimate TSB at high values. The maximum TSB measurement threshold is limited – no readings above 20 mg/dL (342 µmol/L) are possible. Positive test results require confirmation through TSB measurement.

### Icterometers

Icterometers facilitate visual assessment of jaundice severity and reduce interobserver variability. They enable earlier and appropriate referrals from community settings or health centres to higher-level facilities with capacity for bilirubin testing and phototherapy.

#### BiliStrip™

The BiliStrip™ is a simple, inexpensive two (or three)-colour icterometer to assist mothers and community workers in identifying jaundice and deciding when to seek care. Colours are standardised to 5 mg/dL, 10 mg/dL and 15 mg/dL (85 µmol/L, 170 µmol/L and 235 µmol/L) plasma levels. The infant’s nose is pressed with a finger to blanch the skin, and the colour is compared with the BiliStrip™. In a study of nearly 2500 newborns, mothers (using a two-colour BiliStrip™) accurately identified infants requiring phototherapy in all but one infant who needed phototherapy.^10^ For neonates requiring phototherapy, the sensitivity was good (96%) but specificity poor (23%) compared to TSB, resulting in a positive likelihood ratio (+LR) of only 1.2.

#### Bili-ruler™

**FIGURE 2:**
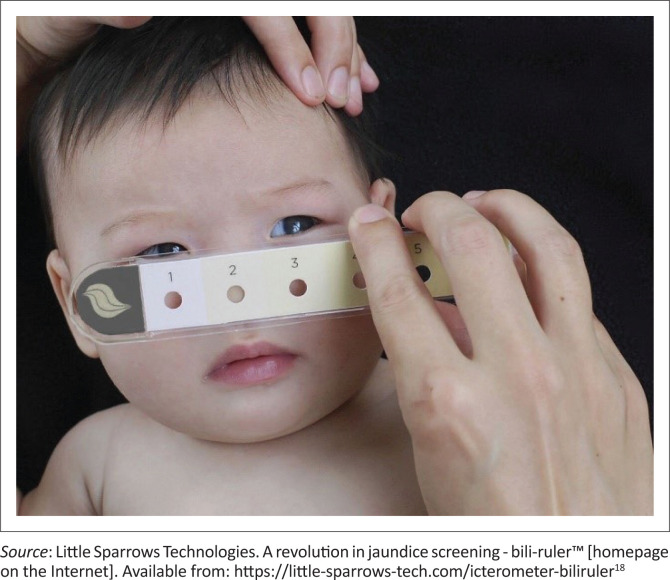
Bili-ruler icterometer.

The Bili-ruler™ has six colour strips developed by processing digital colour photographs of newborn infants with different TSB levels. A healthcare provider matches the colour of the infant’s blanched skin at the nose with that closest to the Bili-ruler™. In one study, the sensitivity and +LR for TSB readings ≥ 13 mg/dL (222 µmol/L) were 82% and 3.2, respectively.^12^ Interrater reliability was high (Figure 2).

### Smartphone digital camera-based bilirubin estimation

A novel approach for estimating TSB levels and identifying hyperbilirubinaemia involves using a mobile phone camera in combination with advanced digital image processing techniques. These applications use feature extraction and machine learning regression to estimate the TSB level. Various smartphone applications (apps) have been created to simplify neonatal jaundice screening. These apps rely on detecting the yellowish discolouration of a newborn’s skin or sclerae. Smartphone apps offer distinct advantages in terms of cost and accessibility to parents and caregivers. They can eliminate the cost, anxiety and hassle of blood draws and extra outpatient hospital visits. Moreover, the technology offers a method of continuous screening, that is repeat measurements can be undertaken easily.

#### BiliCam

**FIGURE 3:**
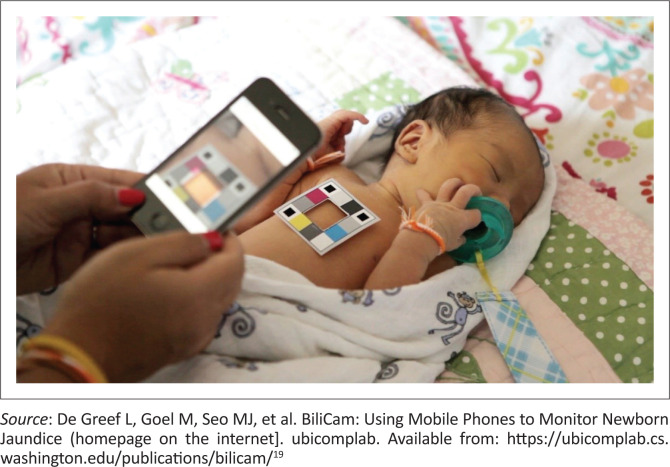
BiliCam moblie phone application.

Using commodity smartphones, the BiliCam app obtains images of the skin overlying a newborn’s sternum against a custom, low-cost colour calibration card. The system uploads the images to a server where, following quality assurance checks, the programme estimates the bilirubin level. The results are communicated back to the user and a course of action recommended. No additional hardware is required. At seven sites across the United States, the BiliCam bilirubin differed from TSB by an average of 0.17 µmol/L ± 31 µmol/L, with 92% of BiliCam bilirubin values falling within 51 µmol/L of the corresponding TSB levels. It achieved 100% sensitivity, 76% specificity and +LR of 4.2 in identifying TSB levels ≥ 291 µmol/L^13^ (Figure 3).

In Norway, a study reported a mean (± standard deviation [s.d.]) difference of −0.2 µmol/L ± 41 µmol/L between *Picterus* image estimates and TSB.^14^ It achieved 100% sensitivity, 69% specificity and +LR of 3.1 in identifying TSB levels > 250 µmol/L.^14^ In contrast, in Singaporean infants, *Biliscan* obtained moderate correlation and mediocre agreement between it and TSB, with a sensitivity of 77%, specificity of 71% and +LR of 2.7 for jaundice requiring phototherapy.^15^

Smartphone-based bilirubin estimation research is currently popular but with no commercial products available yet. Although useful for screening they are not a viable alternative to TcB presently. Additional developments, such as adaptation for darker skin tones, are necessary before smartphone technology for bilirubin estimation will become ubiquitous in LMIC settings.

## Phototherapy

Neonatal jaundice can be treated with phototherapy, which breaks down bilirubin for easy removal from the body. In RCS, conventional phototherapy is typically administered at an irradiance of 8 µW/cm²/nm – 10 µW/cm²/nm, compared to well-equipped settings where higher levels (30 µW/cm²/nm) are possible. Blue lamps are costly and less accessible than white, fluorescent tubes. Hospitals and clinics in RCS often lack working phototherapy machines and reliable electricity for them. Phototherapy quality may deteriorate when infants share units or are incorrectly positioned or exposed.

### Optimising conventional (non-light-emitting diodes) phototherapy

A 2011 Cochrane review found that using light-emitting diodes (LED) and non-LED (fluorescent tube or halogen bulb) phototherapy resulted in similar TSB reduction rates.^16^ Arrays of regular white and daylight fluorescent tubes display acceptable efficiency if appropriately deployed. Irradiances of 20 µW/cm^2^/nm to 40 µW/cm^2^/nm can be attained, particularly if the phototherapy unit is covered by reflecting material such as white 100% cotton cloths or white plastic covers. This simple addition can significantly reduce the duration of phototherapy required.^17^

### Light-emitting diodes phototherapy

The cost of LED phototherapy has reduced markedly in recent years, allowing RCS to replace fluorescent tubes and halogen bulbs with LED as the preferred light source for treating hyperbilirubinaemia, thereby creating the opportunity to consistently provide high-quality phototherapy. Light-emitting diodes phototherapy bulbs typically have a 40 000-hour or longer lifespan (eliminating the need for frequent replacement of bulbs), produce minimal heat and use little energy, making it an affordable technology for RCS.

### Intermittent phototherapy

A 2023 systematic review, which included 12 randomised controlled trials, compared intermittent phototherapy with continuous phototherapy by any method and at any dose and duration.^20^ It identified little or no difference between the two interventions in the rate of decline of bilirubin in term jaundiced infants. Continuous phototherapy was more effective in preterm infants. This lack of difference, if validated by further studies, may have implications for RCS where daytime therapy could be offered at health centres and infants allowed to return home at night before returning the next day.

### Home-based phototherapy

Home-based phototherapy is advocated in certain settings. Its advantages include avoidance of prolonged hospital stays, reducing hospitalisation costs and promoting mother–infant bonding. No high-quality evidence currently exists to support or discourage this practice in term infants.

### Non-useful strategies

#### Turning

Keeping infants supine during phototherapy is as effective as turning them periodically, indicating that the most likely site of bilirubin photo-conversion is in capillaries.^21^

#### Intravenous fluid supplementation

Intravenous fluid supplementation can decrease TSB levels from 8 h to 36 h of life and reduce the need for exchange transfusion but does not influence the duration of phototherapy.^22^ It does not affect important clinical outcomes such as bilirubin encephalopathy, kernicterus or cerebral palsy in healthy, term newborn infants with unconjugated hyperbilirubinaemia requiring phototherapy.^23^

## Sunlight

Sunlight encompasses the light wavelengths found in phototherapy machines but also carries detrimental ultraviolet light and infrared radiation. Extended exposure to sunlight may result in sunburn, skin harm and hypo- and hyper-thermia.^24^ A systematic review suggested that sunlight may be an effective adjunct to conventional phototherapy in RCS settings but should not be offered alone. It may be advisable to filter sunlight to eliminate harmful ultraviolet rays and regularly monitor the temperature of infants exposed to sunlight for safety reasons.^24^ In Nigeria, a transparent polycarbonate room lined with commercial tinting film to provide filtered sunlight phototherapy to infants was found to be safe and no less efficacious than intensive phototherapy for the treatment of moderate-to-severe neonatal hyperbilirubinaemia in near- and full-term infants.^25^

## Adjuvant therapy

New treatments for neonatal jaundice help clear bilirubin faster, reduce phototherapy time and lower the need for exchange transfusions. Practices such as infant massage,^26^ and medications such as fenofibrate,^27^ zinc sulphate,^28^ and ursodeoxycholic acid^29^ can help, although some may need a few days to show benefit. High costs and limited or no utility to prevent bilirubin-induced neurological dysfunction may constrain use in RCS.

## Conclusion

Numerous promising interventions aimed at enhancing the detection of severe hyperbilirubinaemia and optimising phototherapy administration hold the potential to transform the nature of neonatal jaundice management in RCS significantly. While some of the most captivating innovations are currently still in the testing phase, their imminent commercialisation and broad accessibility at affordable prices are on the horizon.

## References

[1] Bhutani VK, Stark AR, Lazzeroni LC, et al. Predischarge screening for severe neonatal hyperbilirubinemia identifies infants who need phototherapy. J Pediatr. 2013;162(3):477.e1–82.e1. 10.1016/j.jpeds.2012.08.022 23043681

[2] Lawn JE, Blencowe H, Oza S, et al. Every newborn: Progress, priorities, and potential beyond survival. Lancet. 2014;384(9938):189–205. 10.1016/S0140-6736(14)60496-7 24853593

[3] Olusanya BO, Ogunlesi TA, Slusher TM. Why is kernicterus still a major cause of death and disability in low-income and middle-income countries? Arch Dis Child. 2014;99(12):1117–1121. 10.1136/archdischild-2013-305506 25123403

[4] Shapiro A, Mtenthaonga P, Mjumira R, et al. Design and field evaluation of a lateral flow cassette device for point-of-care bilirubin measurement. PLoS Glob Public Health. 2023;3(8):e0002262. 10.1371/journal.pgph.0002262 37552665 PMC10409260

[5] Kamineni B, Tanniru A, Vardhelli V, et al. Accuracy of bilistick (a point-of-care device) to detect neonatal hyperbilirubinemia. J Trop Pediatr. 2020;66(6):630–636. 10.1093/tropej/fmaa026 32433770

[6] Rice 360. BiliSpec bilirubin measuring device [homepage on the Internet]. 2023 [cited 2023 Dec 16]. Available from: https://rice360.wixsite.com/rice360/bilispec

[7] Okwundu C, Bhutani VK, Smith J, Esterhuizen TM, Wiysonge C. Predischarge transcutaneous bilirubin screening reduces readmission rate for hyperbilirubinaemia in diverse South African newborns: A randomised controlled trial. S Afr Med J. 2020;110(3):249–254. 10.7196/SAMJ.2020.v110i3.14186 32657704

[8] Olusanya BO, Imosemi DO, Emokpae AA. Differences between transcutaneous and serum bilirubin measurements in Black African Neonates. Pediatrics. 2016;138(3):e20160907. 10.1542/peds.2016-0907 27577578

[9] Okwundu CI, Olowoyeye A, Uthman OA, et al. Transcutaneous bilirubinometry versus total serum bilirubin measurement for newborns. Cochrane Database Syst Rev. 2023;5(5):Cd012660. 10.1002/14651858.CD012660.pub2 37158489 PMC10167941

[10] Olusanya BO, Slusher TM, Imosemi DO, Emokpae AA. Maternal detection of neonatal jaundice during birth hospitalization using a novel two-color icterometer. PLoS One. 2017;12(8):e0183882. 10.1371/journal.pone.0183882 28837635 PMC5570328

[11] Bilimetric. The Bilistick^®^ System [homepage on the Internet]. [cited 2024 Feb 22]. Available from: https://www.bilimetrix.net/bilistick-system/

[12] Lee AC, Folger LV, Rahman M, et al. A Novel icterometer for hyperbilirubinemia screening in low-resource settings. Pediatrics. 2019;143(5):e20182039. 10.1542/peds.2018-2039 30952779

[13] Taylor JA, Stout JW, De Greef L, et al. Use of a smartphone app to assess neonatal jaundice. Pediatrics. 2017;140(3):e20170312. 10.1542/peds.2017-0312 28842403 PMC5574723

[14] Aune A, Vartdal G, Bergseng H, Randeberg LL, Darj E. Bilirubin estimates from smartphone images of newborn infants’ skin correlated highly to serum bilirubin levels. Acta Paediatr. 2020;109(12):2532–2538. 10.1111/apa.15287 32267569

[15] Ngeow AJH, Tan MG, Dong X, et al. Validation of a smartphone-based screening tool (Biliscan) for neonatal jaundice in a multi-ethnic neonatal population. J Paediatr Child Health. 2023;59(2):288–297. 10.1111/jpc.16287 36440650

[16] Kumar P, Chawla D, Deorari A. Light-emitting diode phototherapy for unconjugated hyperbilirubinaemia in neonates. Cochrane Database Syst Rev. 2011;2011(12):Cd007969. 10.1002/14651858.CD007969.pub2 22161417 PMC6885069

[17] Lee Wan Fei S, Chew KS, Pawi S, et al. Systematic review of the effect of reflective materials around a phototherapy unit on bilirubin reduction among neonates with physiologic jaundice in developing countries. J Obstet Gynecol Neonatal Nurs. 2018;47(6):795–802. 10.1016/j.jogn.2018.07.008 30172596

[18] Little Sparrows Technologies. A revolution in jaundice screening - bili-ruler™ [homepage on the Internet]. [cited 2024 Feb 22]. Available from: https://little-sparrows-tech.com/icterometer-biliruler

[19] De Greef L, Goel M, Seo MJ, et al. BiliCam: Using Mobile Phones to Monitor Newborn Jaundice (homepage on the internet]. [cited 2024 Feb 22]. ubicomplab. Available from: https://ubicomplab.cs.washington.edu/publications/bilicam/

[20] Gottimukkala SB, Lobo L, Gautham KS, Bolisetty S, Fiander M, Schindler T. Intermittent phototherapy versus continuous phototherapy for neonatal jaundice. Cochrane Database Syst Rev. 2023;3(3):Cd008168. 10.1002/14651858.CD008168.pub2 36867730 PMC9979775

[21] Lee Wan Fei S, Abdullah KL. Effect of turning vs. supine position under phototherapy on neonates with hyperbilirubinemia: A systematic review. J Clin Nurs. 2015;24(5–6):672–682. 10.1111/jocn.12712 25319831

[22] Gu J, Zhu Y, Zhao J. The efficacy of intravenous fluid supplementation for neonatal hyperbilirubinemia: A meta-analysis of randomized controlled studies. J Matern Fetal Neonatal Med. 2021;34(21):3580–3585. 10.1080/14767058.2019.1688295 31736410

[23] Lai NM, Ahmad Kamar A, Choo YM, Kong JY, Ngim CF. Fluid supplementation for neonatal unconjugated hyperbilirubinaemia. Cochrane Database Syst Rev. 2017;8(8):Cd011891. 10.1002/14651858.CD011891.pub2 28762235 PMC6483308

[24] Horn D, Ehret D, Gautham KS, Soll R. Sunlight for the prevention and treatment of hyperbilirubinemia in term and late preterm neonates. Cochrane Database Syst Rev. 2021;7(7):Cd013277. 10.1002/14651858.CD013277.pub2 34228352 PMC8259558

[25] Slusher TM, Vreman HJ, Brearley AM, et al. Filtered sunlight versus intensive electric powered phototherapy in moderate-to-severe neonatal hyperbilirubinaemia: A randomised controlled non-inferiority trial. Lancet Glob Health. 2018;6(10):e1122–e1131. 10.1016/S2214-109X(18)30373-5 30170894

[26] Zhang M, Wang L, Wang Y, Tang J. The influence of massage on neonatal hyperbilirubinemia: A meta-analysis of randomized controlled trials. J Matern Fetal Neonatal Med. 2019;32(18):3109–3114. 10.1080/14767058.2018.1455183 29631455

[27] Shabo SK, Gargary KH, Erdeve O. Indirect neonatal hyperbilirubinemia and the role of fenofibrate as an adjuvant to phototherapy. Children. 2023;10(7):1192. 10.3390/children10071192 37508689 PMC10378335

[28] Faal G, Khatib Masjedi H, Sharifzadeh G, Kiani Z. Efficacy of zinc sulfate on indirect hyperbilirubinemia in premature infants admitted to neonatal intensive care unit: A double-blind, randomized clinical trial. BMC Pediatr. 2020;20(1):130. 10.1186/s12887-020-02025-9 32192467 PMC7081620

[29] Kuitunen I, Kiviranta P, Sankilampi U, Renko M. Ursodeoxycholic acid as adjuvant treatment to phototherapy for neonatal hyperbilirubinemia: A systematic review and meta-analysis. World J Pediatr. 2022;18(9):589–597. 10.1007/s12519-022-00563-z 35689782 PMC9376150

